# *Bacillus anthracis* Spore Entry into Epithelial Cells Is an Actin-Dependent Process Requiring c-Src and PI3K

**DOI:** 10.1371/journal.pone.0011665

**Published:** 2010-07-20

**Authors:** Qiong Xue, Sarah A. Jenkins, Chunfang Gu, Emanuel Smeds, Qing Liu, Ranga Vasan, Brooke H. Russell, Yi Xu

**Affiliations:** 1 Center for Infectious and Inflammatory Diseases, Institute of Biosciences and Technology, Texas A&M Health Science Center, Houston, Texas, United States of America; 2 Graduate School of Biomedical Sciences, University of Texas Health Science Center, Houston, Texas, United States of America; 3 Department of Thoracic and Cardiovascular Surgery, M.D. Anderson Cancer Center, Houston, Texas, United States of America; University of Minnesota, United States of America

## Abstract

Dissemination of *Bacillus anthracis* from the respiratory mucosa is a critical step in the establishment of inhalational anthrax. Recent *in vitro* and *in vivo* studies indicated that this organism was able to penetrate the lung epithelium by directly entering into epithelial cells of the lung; however the molecular details of *B. anthracis* breaching the epithelium were lacking. Here, using a combination of pharmacological inhibitors, dominant negative mutants, and colocalization experiments, we demonstrated that internalization of spores by epithelial cells was actin-dependent and was mediated by the Rho-family GTPase Cdc42 but not RhoA or Rac1. Phosphatidylinositol 3-kinase (PI3K) activity was also required as indicated by the inhibitory effects of PI3K inhibitors, wortmannin and LY294002, and a PI3K dominant negative (DN) mutant Δp85α. In addition, spore entry into epithelial cells (but not into macrophages) required the activity of Src as indicated by the inhibitory effect of Src family kinase (SFK) inhibitors, PP2 and SU6656, and specific siRNA knockdown of Src. Enrichment of PI3K and F-actin around spore attachment sites was observed and was significantly reduced by treatment with SFK and PI3K inhibitors, respectively. Moreover, *B. anthracis* translocation through cultured lung epithelial cells was significantly impaired by SFK inhibitors, suggesting that this signaling pathway is important for bacterial dissemination. The effect of the inhibitor on dissemination *in vivo* was then evaluated. SU6656 treatment of mice significantly reduced *B. anthracis* dissemination from the lung to distal organs and prolonged the median survival time of mice compared to the untreated control group. Together these results described a signaling pathway specifically required for spore entry into epithelial cells and provided evidence suggesting that this pathway is important for dissemination and virulence *in vivo*.

## Introduction

Inhalational anthrax is a life-threatening infection initiated by pulmonary exposure to *Bacillus anthracis* spores. The pathogen then disseminates away from the lung to establish a systemic infection. The systemic spread is thought to come from hematogenous sources; however, how *B. anthracis* disseminates from the lung, the initial entry site, to the blood remains poorly understood.

Although *B. anthracis* is primarily an extracellular pathogen, studies from multiple groups have indicated that an intracellular stage is necessary for the pathogen to breach the lung epithelial barrier [1], [2], [3], [4]. Mice can be protected by immunization with inactivated spores. The protection was found to be from cellular rather than humoral immunity, further highlighting the importance of an intracellular stage in the establishment of anthrax infections [5]. In the lung, spores encounter three major types of cells, epithelial cells in the alveoli and small airway, resident alveolar macrophages (AMs), and lung dendritic cells (LDCs). AMs and LDCs have been indicated to play roles in the dissemination process by first engulfing spores and then carrying them to regional lymph nodes [2], [3]. Spores germinate inside the phagocytes, replicate and eventually escape from them via an undefined mechanism. Another strategy often used by pathogens to breach mucosal barriers is by entering into non-phagocytic host cells and then escaping from them. Recent studies suggested that *B. anthracis* spores may use this strategy as well [1], [4]. Spores of *B. anthracis* can be internalized by polarized A549 cells (human alveolar type II-like epithelial cells) and primary human small airway epithelial cells (hSAECs) [1], [6]. In addition, substantial amounts of spores were found inside epithelial cells of the lung in mice within hours of inoculation [4], indicating that spore entry into lung epithelial cells is relevant *in vivo*. Furthermore, *B. anthracis* can cross a barrier of lung epithelial cells in the absence of phagocytes and without compromising the barrier integrity [1]. Spores and vegetative bacilli are also able to survive inside lung epithelial cells [1], in contrast to the finding in macrophages [7], [8], [9]. Thus spore entry into lung epithelial cells appears to be an important early event in the development of inhalational anthrax.

Spore-lung epithelium interactions have also been shown to influence host immune responses. Using a human lung slice model, Chakrabarty *et al*. observed activation of the mitogen-activated protein kinase signaling pathways and increases in the cytokines levels (e.g., IL-6, TNF-α, IL8, MIP-1α/β, and MCP-1) upon exposure to spores. Lung epithelial cells as well as alveolar macrophages were the main sources for the increased cytokines and chemokines [10]. A recent report by Evans *et al*. showed that mice treated with bacterial lysates developed an innate immunity to infections by *B. anthracis* spores. Interestingly, lung epithelial cells not macrophages or neutrophils were responsible for the induced resistance [11]. These results further underscored the importance of spore-epithelium interactions in the pathogenesis of *B. anthracis*. However, prior to this study little information was available regarding the molecular mechanism of spore-epithelium interactions, what factors mediate spore entry into epithelial cells or the biological consequence of disrupting the entry process.

We previously showed that spore germination was not required for internalization by non-phagocytic cells, and that spores of *B. subtilis* were internalized by host cells at a significantly lower frequency than that of *B. anthracis* spores [1], [6]. These results indicated that specific components on *B. anthracis* spores were necessary and sufficient to induce spore entry into non-phagocytic cells. Therefore, in this study we sought to investigate the entry mechanism of wild-type spores by elucidating the cellular components and signaling molecules in epithelial cells required for the internalization process. Using a combination of specific pharmacological inhibitors, dominant negative mutants, colocalization experiments and specific siRNA knockdown, a signaling pathway responsible for mediating the internalization of spores by epithelial cells was uncovered. The importance of this signaling pathway in bacterial dissemination *in vitro* and *in vivo* was also investigated.

## Results

### *B. anthracis* spore internalization by epithelial cells is actin-dependent

**Figure 1:**
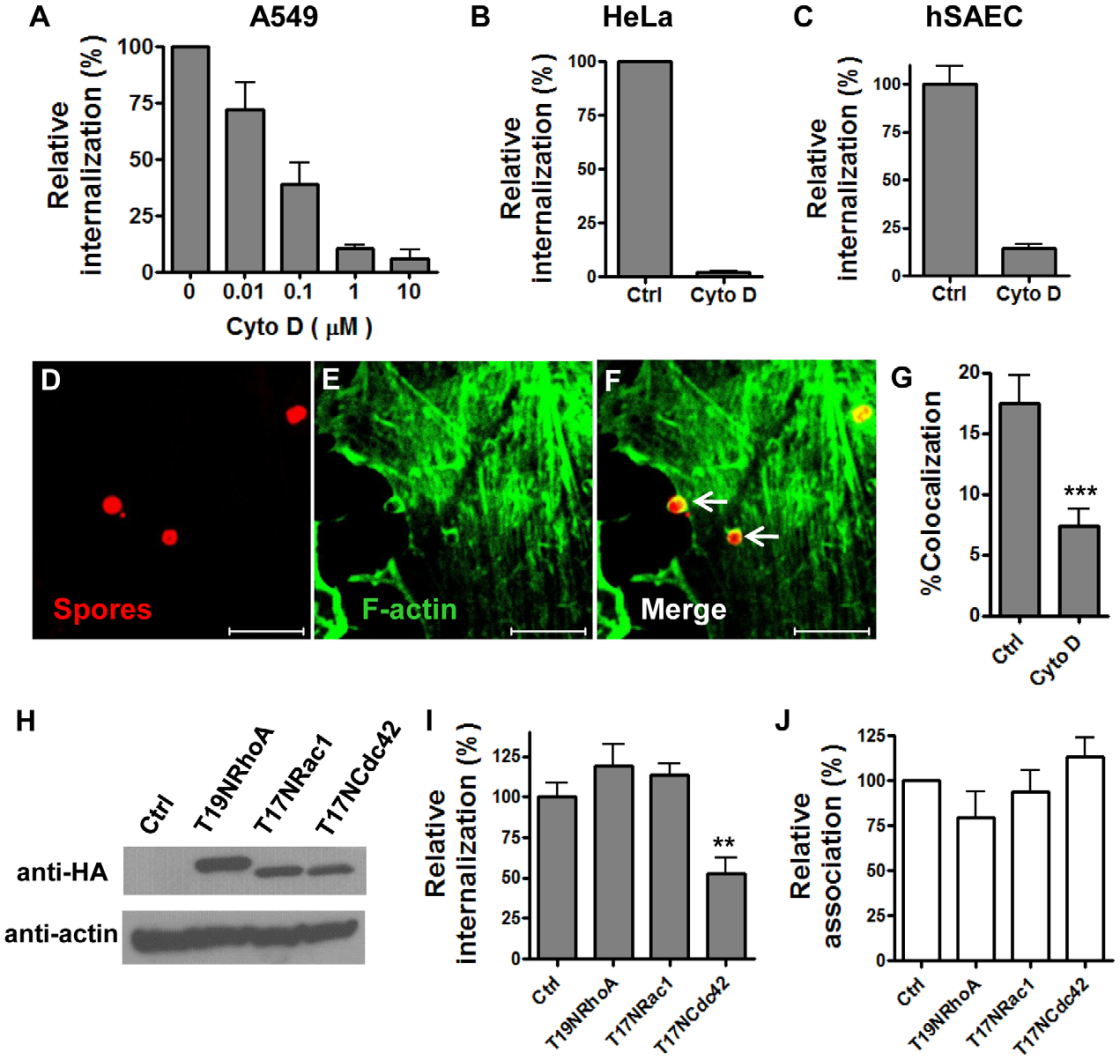
*B. anthracis* spore internalization by epithelial cells requires actin polymerization mediated by Cdc42. **A** – **C**, the effect of cytochalasin D on spore internalization by A549 (**A**), HeLa (**B**) and hSAECs (**C**). Cells were pretreated with different concentrations (0.01 – 10 µM for A549 cells, 1 and 10 µM for HeLa and hSAECs, respectively) of cytochalasin D (Cyto D) or the solvent control (Ctrl) for 1 hr and then performed fluorescent microscopic examination as described in Materials and Methods. Relative internalization (%) is the ratio of intracellular spores vs. total adhered spores, normalized to the solvent control. The results are the mean ± SEM from at least two independent experiments. **D** – **F**, representative images of colocalization of F-actin with spores. A549 cells were incubated with Texas-Red labeled spores and then stained with phalloidin-Alexa Fluor 488. **G**, colocalization of F-actin with spores is inhibited by Cyto D. Cyto D (10 µM) was included in the colocalization examination as described in Materials and Methods. The percentage of spores with enriched F-actin staining vs. total attached spores is denoted as %Colocalization. The results shown are combined from five independent experiments. ***, *p<*0.0001, *t* test. Scale bars represent 5 µm. **H** – **J**, Cdc42 is the primary Rho-family GTPase required for spore uptake. A549 cells were transfected with pcDNA3.1(+) vector control (Ctrl), HA-T19NRhoA (T19NRhoA), HA-T17NRac1 (T17NRac1) or HA-T17NCdc42 (T17NCdc42). The expression of the transfected constructs was verified using western blot analysis 24 hr post transfection (**H**). Dominant negative GTPases were detected by anti-HA antibodies and secondary antibodies as described in Materials and Methods. The actin level was used as a loading control. Spore uptake by (**I**) and adherence to (**J**) transfected cells were determined by gentamicin protection assays 24 hr post transfection as described in Materials and Methods. Relative internalization (%) is the ratio of intracellular spores vs. total spores added, normalized to the solvent control. Relative association (%) is the ratio of associated spores (extracellular adhered + intracellular) vs. total spores added, normalized to the solvent control. The results shown are the mean ± SEM, combined from three independent experiments. **, *p* = 0.0018, *t* test.

We first examined if spore internalization by epithelial cells was dependent on the actin cytoskeleton. Cytochalasin D, an inhibitor of actin polymerization, inhibited spore uptake by A549 cells in a dose-dependent manner (Fig. 1, A). Uptake of spores was nearly abolished in the presence of 10 µM cytochalasin D. Similar results were observed in HeLa cells and hSAECs (Fig. 1, B and C). Cell viability was not affected by cytochalasin D at the concentrations used, as assessed by trypan blue exclusion. Nor was spore viability affected, as determined by plating and colony counts.

We further investigated if spores colocalized with F-actin during entry by fluorescence microscopy. After 30 minutes of incubation, approximately 17.4% of the total attached spores were seen surrounded by enriched F-actin staining (SEM  = 1.9%, n = 6 independent assays) (Fig. 1, D – G), suggesting that there was local activation of actin polymerization or reorganization at these sites. In general, the ratio of internalization spores vs. attached spores under our assay conditions is approximately 1∶10–1∶5. The percentage of attached spores with enriched F-actin is consistent with this ratio. We determined that spores did not emit green fluorescence by themselves by examining spores attached to coverslips in the absence of cells. To rule out the possibility that the colocalization was due to preferential attachment of spores to pre-existing actin-rich patches, we performed the experiment in the presence of cytochalasin D. Colocalization of spores with F-actin was significantly reduced (by ∼61%) in cytochalasin D-treated cells (***, *p<*0.001 compared to no inhibitor), suggesting that there was active polymerization of F-actin at these spore attachment sites (Fig. 1, G). Cytochalasin D did not completely abolish F-actin enrichment around spores. This could be due to the possibility that cytochalasin D prevented short actin filaments from polymerization. However, these short actin filaments could still be recruited to the spore attachment sites, although they were not able to drive the internalization process [12]. Together the above results indicated that spore internalization by epithelial cells required actin polymerization.

### The Rho-family GTPase Cdc42 is required for spore uptake

The Rho family of small GTPases regulates the polymerization and reorganization of the actin cytoskeleton. RhoA, Rac1 and Cdc42 are the three major Rho GTPases. RhoA mainly mediates stress fiber formation, Rac1 lamellipodia and filopodia, and Cdc42 filopodia [13]. We investigated which of the three Rho GTPases was responsible for spore internalization by epithelial cells. T19NRhoA, T17NRac1 and T17NCdc42 are mutants of these GTPases that lack the ability to adopt the active GTP-bound form, but maintain the ability to bind guanine nucleotide exchange factors (GEFs). They are widely used as dominant negative (DN) mutants for the respective proteins [14]. HeLa cells were transfected with plasmids expressing either HA-tagged T19NRhoA, T17NRac1, T17NCdc42 or the vector control, respectively. The expression of the three DN mutant proteins in transfected cells was confirmed by western blot analysis of cell lysates 24 hours post-transfection (Fig. 1, H). Spore internalization was significantly reduced in cells transfected with T17NCdc42, but not in cells transfected with T19NRhoA or T17NRac1 (Fig. 1, I). None of the three DN mutants affected spore adherence to cells (Fig. 1, J), as expected. Transfection efficiency was approximately 80%, as determined by transfecting cells with a GFP expressing plasmid. The relatively moderate inhibition by DN Cdc42 mutant compared to that by cytochalasin D treatment could be due to incomplete transfection and/or incomplete inhibition of the endogenous Cdc42 activity. Similar results were observed in A549 cells transfected with the respective plasmids, *i.e.*, approximately 35% decreases in spore internalization were only observed in A549 cells transfected with T17NCdc42 but not in cells transfected with the other two DN mutants (data not shown). Transfection did not affect cell viability, assessed by trypan blue exclusion. These results indicate that Cdc42, but not Rac1 or RhoA, regulates actin polymerization during spore uptake.

### Internalization of *B. anthracis* spores by epithelial cells requires phosphatidylinositol 3-kinase (PI3K)

**Figure 2:**
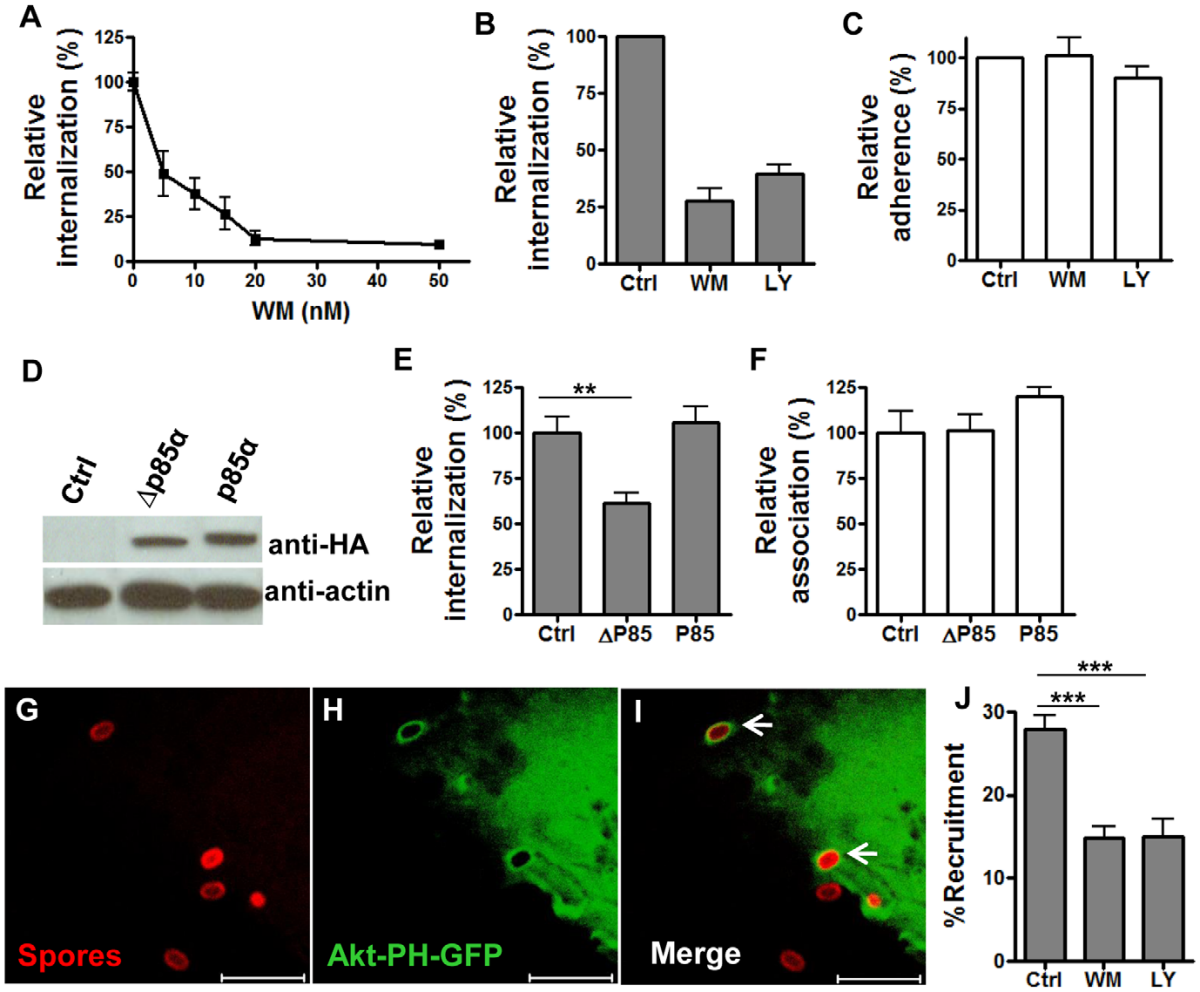
*B. anthracis* spore internalization by epithelial cells requires PI3K activity. **A** – **C**, spore internalization by A549 cells is inhibited by PI3K inhibitors, wortmannin (WM) and LY294002 (LY). Cells were pre-incubated with 0–50 nM WM, 50 µM LY, or the solvent control (Ctrl) for 1 hr and infected with spores in the presence of the inhibitor. Intracellular and extracellular spores were determined by gentamicin protection assay (**A**) and fluorescence microscopic examination (**B** and **C**). Relative internalization (%) is as described in the legend for Fig. 1. Relative adherence (%) is the ratio of extracellular bound spores vs. total fields counted, normalized to the control. The gentamicin protection result is the mean ± SEM from a representative experiment. The fluorescence microscopic results are combined from at least two independent experiments. **D** – **F**, spore internalization by A549 cells is inhibited by dominant negative p85. A549 cells were transfected with SRα vector control (Ctrl), HA-Δp85α (Δp85α) and HA-p85α (p85α). The expression of HA-Δp85α and HA-p85α in transfected cells was analyzed by western blot using anti-HA antibodies and appropriate secondary antibodies 24 hr post transfection (**D**). Spore internalization by (**E**) and association with (**F**) transfected cells were determined using gentamicin protection assays 24 hr post transfection. The results are the mean ± SEM, combined from three independent experiments. ***, *p*<0.001; **, *p*<0.01, *t* test. **G** – **I**, PI3K is rapidly recruited and activated at spore entry sites. A549 cells were transfected with Akt-PH-GFP, incubated with Texas Red-labeled spores for 8 min and processed for confocal examination. Representative images are shown. Arrows indicate the spore attachment sites where Akt-PH-GFP was recruited. Scale bars represent 5 µm. **J**, Quantitation of Akt-PH-GFP recruitment at spore attachment sites in the absence (Ctrl) or presence of the PI3K inhibitors. WM (100 nM) or LY (50 µM) was included in the colocalization assay. The percentage of spores surrounded with enhanced green fluorescence vs. total attached spores is denoted as %Recruitment. The results shown are combined from four independent experiments. ***, *p<*0.001, *t* test.

PI3K is required for the internalization of a number of bacteria by host non-phagocytic cells. We tested the effect of different concentrations (0–50 nM) of wortmannin, a specific PI3K inhibitor, on spore internalization by A549 cells. A dose-dependent inhibition was observed (Fig. 2, A). The IC_50_ was calculated to be ∼6–10 nM (non-linear regression curve fit, GraphPad Prism software), consistent with the reported IC_50_ of wortmannin for PI3K [15]. The effect was not specific to A549 cells. Spore uptake by HeLa cells was also dramatically reduced (∼80%) by wortmannin (100 nM) (Fig. S1, A). LY294002 inhibits PI3K enzymatic activities by a mechanism distinct from that of wortmannin [15]. The effect of LY294002 was also tested. LY294002 had a similar effect on spore internalization as that of wortmannin, ∼60% reduction on spore internalization by A549 cells (Fig. 2, B) and ∼70% by HeLa cells at the concentration used (Fig. S1, A). As expected, no significant effect on spore adherence to A549 (Fig. 2, C) or HeLa cells (Fig. S1, B) by either inhibitor was observed. The fact that both inhibitors caused significant reduction on spore internalization strongly suggests that PI3K activity is required for spore internalization. Neither wortmannin nor LY294002 affected the viability of cells or spores.

As PI3Ks are also involved in other cellular processes such as intracellular vesicle trafficking, we tested if the reduction of intracellular bacteria in inhibitor treated cells was due to alterations in some intracellular processes post spore entry. Spores were allowed to be taken up by A549 cells in the absence of the inhibitor and then treated with wortmannin. No significant difference in the number of intracellular bacteria was observed between wortmannin-treated cells and the control cells (data not shown), suggesting that the reduction of intracellular bacteria caused by the PI3K inhibitors was due to impaired spore entry.

There are three classes of PI3Ks, among which class I PI3Ks are involved in regulating the actin cytoskeleton. This class is further divided into two sub-groups, IA and IB. Class IA enzymes are ubiquitously expressed and are made up of a 110 kDa catalytic subunit (p110) and an adaptor/regulatory subunit, the most abundant of which in mammalian cells is p85α [16]. Class IB PI3K is primarily expressed in white blood cells. We tested if spore entry into epithelial cells was mediated by a class IA PI3K. A dominant negative construct for class IA PI3Ks, Δp85α, was employed for this purpose. This mutant lacks the binding site for the catalytic p110 subunit and therefore cannot recruit p110 upon activation [17]. Plasmid constructs of HA-tagged Δp85α, wild-type p85α, or SRα (vector) were transfected into A549 and HeLa cells, respectively. Transfection efficiency was approximately 40% for A549 cells and 80% for HeLa cells, estimated by transfecting cells with a GFP-expressing plasmid. Expression of HA-Δp85α and HA-p85α in transfected cells was confirmed by western blot analysis of cell lysates 24 hours post-transfection using anti-HA antibodies (Fig. 2, D and Fig. S1, C). The mutant migrated slightly faster than wild-type p85α since it lacked the p110 binding site and had a smaller molecular weight. Analysis of spore internalization showed that expression of Δp85α significantly decreased the spore internalization frequency (by ∼45%) in both A549 (Fig. 2, E, **, *p*<0.01) and HeLa cells (Fig. S1, D, ***, *p*<0.001) when compared to cells transfected with the vector control. The decrease (∼45%) in spore internalization caused by Δp85α expression was less dramatic than that by the two PI3K inhibitors. This was likely due to the moderate transfection efficiency, competition between Δp85α and the endogenous p85α, and the presence of other regulatory subunits of PI3Ks, which could potentially compensate for p85α. Over expression of exogenous wide-type p85α did not increase spore internalization, possibly because the amount of endogenous adaptors was sufficient to mediate spore internalization (Fig. 2, E and Fig. S1, D). Spore adherence to cells was not affected by Δp85α expression (Fig. 2, F and Fig. S1, E). Neither HA-Δp85α nor HA-p85α expression affected cell viability as determined by trypan blue exclusion. Altogether, the above results indicate that internalization of *B. anthracis* spores by epithelial cells requires PI3K activity, primarily the activity of a class IA PI3K.

Upon activation, class I PI3Ks phosphorylate PtdIns(4,5)*P*_2_ to PtdIns(3,4,5)*P*_3_, which binds to the PH domain of downstream effectors such as the serine/threonine protein kinase Akt/PKB [18]. In order to examine PI3K recruitment and activation, a construct containing the Akt-PH domain fused to a GFP gene (Akt-PH-GFP) was used as a molecular probe for the PI3K product PtdIns(3,4,5)*P*_3_ [19]. A549 cells transfected with the Akt-PH-GFP construct were serum-starved and then incubated with Texas Red-labeled *B. anthracis* spores. Approximately 28.0% of attached spores recruited Akt-PH-GFP within minutes of incubation (SEM  = 1.6%, n = 6 independent assays) (Fig. 2, G – J). Also, significantly less Akt-PH-GFP recruitment was observed (***, *p<*0.001 compared to no inhibitor) in cells treated with wortmannin or LY294002 (Fig. 2, J), suggesting that the recruitment was due to PI3K activation. The incomplete inhibition of Akt-PH recruitment by the inhibitors has been previously reported [20], [21]. Together, these results further confirm that a class IA PI3K is recruited and activated during spore internalization.

### c-Src, a member of the Src family protein tyrosine kinases, is required for the internalization of *B. anthracis* spores by epithelial cells

**Figure 3:**
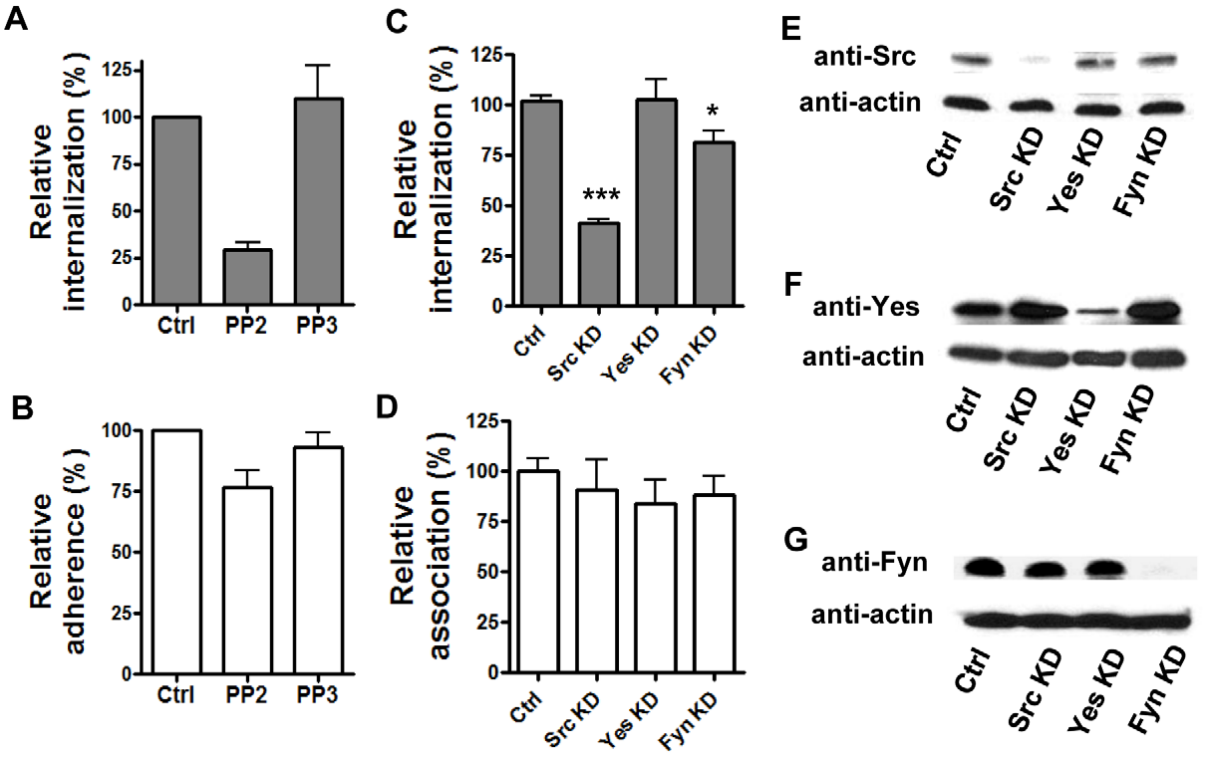
Spore internalization by epithelial cells requires the protein tyrosine kinase Src. **A** and **B**, spore internalization by A549 cells is inhibited by PP2 but not PP3. Cells were pre-treated with PP2 (10 µM), PP3 (10 µM) or solvent control (Ctrl) for 1 hr and then incubated with labeled 7702 spores in the presence of the inhibitors. Intracellular and extracellular spores were quantified by fluorescence microscopic examination. Relative internalization (**A**) and relative adherence (**B**) were calculated as the number of intracellular and extracellular adhered spores vs. total fields counted, respectively, and were normalized to the control. The results are the mean ± SEM from at least 2 independent assays. **C** – **G**, knockdown of c-Src by siRNA significantly reduces spore internalization. A549 cells were transfected with 50 nM siRNA for Src (Src KD), Yes (Yes KD), Fyn (Fyn KD) or control siRNA (Ctrl). Spore internalization (**C**) and adherence (**D**) were determined by gentamicin protection assays 48 hr post transfection. The results were from three independent experiments. ***, *p<*0.001; *, *p* = 0.0165, Student's *t* test, normalized to the control. The knockdown of protein levels in transfected cells was determined by western blot using specific antibodies against Src (**E**), Yes (**F**) and Fyn (**G**).

To determine if Src family protein tyrosine kinase (SFK) activity was required for spore internalization by epithelial cells, PP2, a specific SFK inhibitor and its negative control compound, PP3, were used to treat A549 cells. Spore internalization was inhibited by PP2 (∼70%) but not by PP3 (Fig. 3, A). Spore adherence to A549 cells was not affected by either PP2 or PP3 (Fig. 3, B). SU6656, an SFK inhibitor that has a narrower set of targets than PP2, was also tested. SU6656 at a concentration of 50 µM significantly inhibited spore entry into A549 cells by approximately 70% (data not shown). None of the compounds affected the viability of cells or spores. Next we tested the effect of PP2 on spore uptake by epithelial cells of different origin and spore phagocytosis by macrophages. PP2 inhibited spore uptake by HeLa, hSAECS, and MLE15 (a murine lung epithelial cell line) cells, suggesting that SFK activity is required for spore internalization by epithelial cells from different origins. In contrast the same concentration of PP2 did not affect spore phagocytosis by RAW264.7 macrophages or murine primary peritoneal macrophages (Fig. S2, A), suggesting that SFK activity is specifically required for spore entry into epithelial cells. It was recently reported that PI3K activity was involved in spore phagocytosis by macrophages [22]. We tested the effect of LY294002 and found that spore phagocytosis in fact was inhibited by LY294002 (Fig. S2, B), consistent with the previous report [22].

Of the nine Src family tyrosine kinases, c-Src, c-Yes and Fyn are ubiquitously expressed, while others are primarily expressed in cells of the hematopoietic lineage [23]. Therefore, these three kinases are likely candidates involved in spore internalization. We first tested murine embryonic fibroblasts (MEFs) from *src-/-yes-/-fyn-/-* triple knockout mice (obtained from ATCC). Spore internalization by the triple knockout MEFs was ∼80% lower compared to that by wild type MEFs (data not shown). To determine which one of the three kinases was required for spore internalization by epithelial cells, A549 cells were transfected with specific siRNAs for c-Src, c-Yes and Fyn. Analysis of spore internalization in cells transfected with the different siRNAs indicated that knockdown of c-Src decreased spore internalization by approximately 60% (***, *p<*0.001 compared to the control), whereas knockdown of c-Yes or Fyn had no or only marginal inhibitory effect (Fig. 3, C). Spore adherence was not affected by knockdown of any of the three kinases (Fig. 3, D). Specific knockdown of the targeted protein kinase was confirmed using western blot analysis (Fig. 3, E – G). These results indicate that c-Src is the primary SFK involved in spore uptake by epithelial cells.

### Src and PI3K function in the same signaling pathway to mediate spore internalization

**Figure 4:**
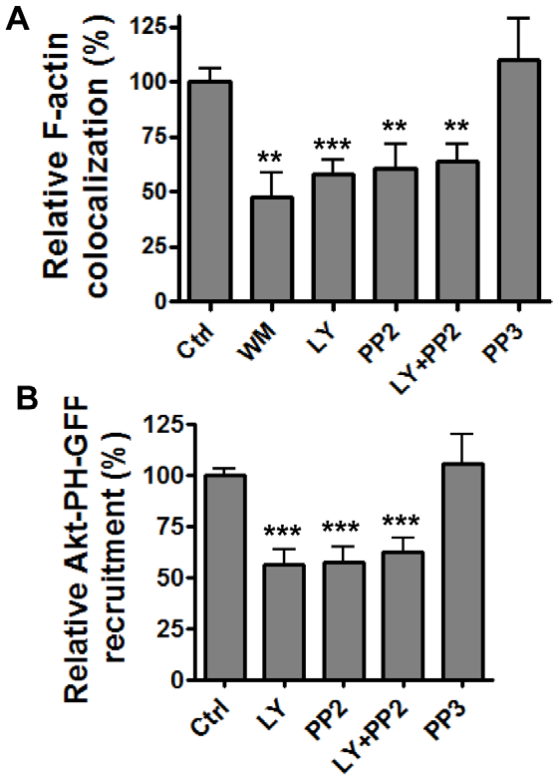
Src and PI3K act in a linear signaling pathway. **A**, F-actin colocalization with spores is reduced in the presence of WM, LY and PP2. A549 cells were pre-incubated with WM (100 nM), LY (50 µM), PP2 (10 µM), PP3 (10 µM), LY (50 µM) + PP2 (10 µM) or solvent control for 1 hr and then incubated with Texas Red-labeled 7702 spores in the presence of the respective inhibitors for 30 min and then stained for F-actin. The samples were examined using confocal fluorescence microscopy. Relative F-actin colocalization is the ratio of spores that associated with enriched F-actin staining vs. total spores that attached to the cells, normalized to the solvent control. **B**, Akt-PH-GFP recruitment is reduced in the presence of LY and PP2. A549 cells transfected with Akt-PH-GFP were pre-incubated with LY (50 µM), PP2 (10 µM), PP3 (10 µM), LY (50 µM) + PP2 (10 µM) or solvent control for 1 hr and then incubated with labeled spores in the presence of the respective inhibitors for 8 min. Relative Akt-PH-GFP recruitment is the ratio of spores that recruited Akt-PH-GFP vs. total spores that attached to the cells, normalized to the solvent control. The results are the mean ± SEM from at least three experiments. ***, *p*<0.001; **, *p*<0.01, *t* test.

To understand the connection between the different signaling molecules elucidated above, we first investigated whether PI3K and c-Src were required for actin polymerization during spore entry. F-actin enrichment around spore attachment sites was significantly reduced in cells treated with the PI3K inhibitors, wortmannin and LY294002, and the SFK inhibitor, PP2, but not in PP3-treated cells (Fig. 4, A). None of these inhibitors disrupted the actin cytoskeleton at the concentration tested as judged by the phalloidin staining pattern in uninfected cells. These results suggested that both PI3K and c-Src are involved in regulating actin polymerization during spore entry.

PI3K and SFKs can act in the same signaling pathway or in independent pathways to transduce signals to downstream effectors leading to the activation of actin cytoskeleton. Therefore, we investigated the connection between PI3K and c-Src in mediating spore uptake by epithelial cells. If PI3K and c-Src function in the same signaling pathway, treating cells with inhibitors for both PI3K and c-Src simultaneously should have the same effect on spore internalization as treating cells with individual inhibitor alone. On the other hand, if they function in independent pathways, inhibition of both kinases would have a synergistic effect compared to inhibition of the individual kinase. The results showed that treatment of A549 cells with both LY294002 and PP2 caused a similar level of reduction in F-actin enrichment around spores compared to the reductions seen with individual inhibitor only (∼40% reduction; **, *p*<0.01) (Fig. 4, A), suggesting that PI3K and SFKs functioned in the same signaling pathway for spore internalization.

To further investigate the order of PI3K and c-Src activation in the signaling cascade, we examined the recruitment of Akt-PH-GFP upon spore attachment in cells treated with PP2. In PP2 treated cells, the recruitment of Akt-PH-GFP was reduced (***, *p<*0.001 compared to the no inhibitor control) to a similar level as those in cells treated with wortmannin or LY294002 (Fig. 4, B). Treatment of cells with both PP2 and LY294002 did not cause any further reduction in Akt-PH-GFP recruitment (∼40%, ***, *p<*0.001) compared to cells treated with the individual inhibitor (Fig. 4, B). PP3 had no effect on Akt-PH-GFP recruitment. These results suggest that PI3K likely act downstream of c-Src in the signaling pathway involved in spore internalization by epithelial cells.

### *B. anthracis* translocation across a lung epithelial barrier requires Src activity

**Figure 5:**
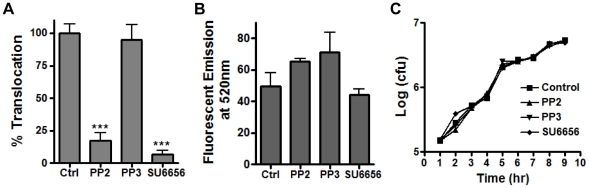
Src activity is required for *B. anthracis* translocation through an A549 cell barrier. A549 cells grown in transwell inserts were pre-treated with PP2 (10 µM), PP3 (10 µM), and SU6656 (50 µM) or solvent (Ctrl) for 1 hr. Spores were then added to the top chambers as described in Materials and Methods. Media was collected from the bottom chambers after 16 hr of incubation. An aliquot of the media was dilution plated to determine bacterial counts (**A**). Another aliquot was used to measure fluorescence emission at 520 nm (FITC-dextran) (**B**). The effect of the inhibitors on bacterial growth was also determined (**C**) as described in Materials and Methods. The results shown are mean ± SEM from at least two independent experiments. ***, *p*<0.001, *t* test.

It was previously shown that *B. anthracis* could translocate across an A549 cell barrier without apparent disruption of the barrier integrity, suggesting that translocation occurred via an intracellular route [1]. We tested if inhibiting Src activity would reduce bacterial translocation. A549 cells grown on transwell inserts were treated with PP2, SU6656, PP3, or no inhibitor. Spores were added to the apical side of A549 cells and incubated in the presence of the inhibitor. Significantly less bacteria were recovered from the bottom chambers of cells treated with PP2 and SU6656 respectively, compared to the control (Fig. 5, A, ***, *p<*0.001). PP3, a negative control compound for PP2, did not have any effect on translocation (Fig. 5, A). The inhibitor treatment did not compromise the A549 cell barrier integrity as assessed by FITC-dextran diffusion from the top to the bottom chambers in the inhibitor-treated and the control wells (Fig. 5, B). To rule out the possibility that the inhibitors affected vegetative growth of *B. anthracis*, which could have resulted in fewer bacteria in the bottom chambers, we examined the effect of the inhibitors on bacterial growth in the tissue culture conditions. No difference was observed (Fig. 5, C). Together, the above results indicate that Src activity is required for *B. anthracis* translocation across a barrier of lung epithelial cells.

### Inhibition of Src activity reduces bacterial dissemination in a mouse model of inhalational anthrax

**Figure 6:**
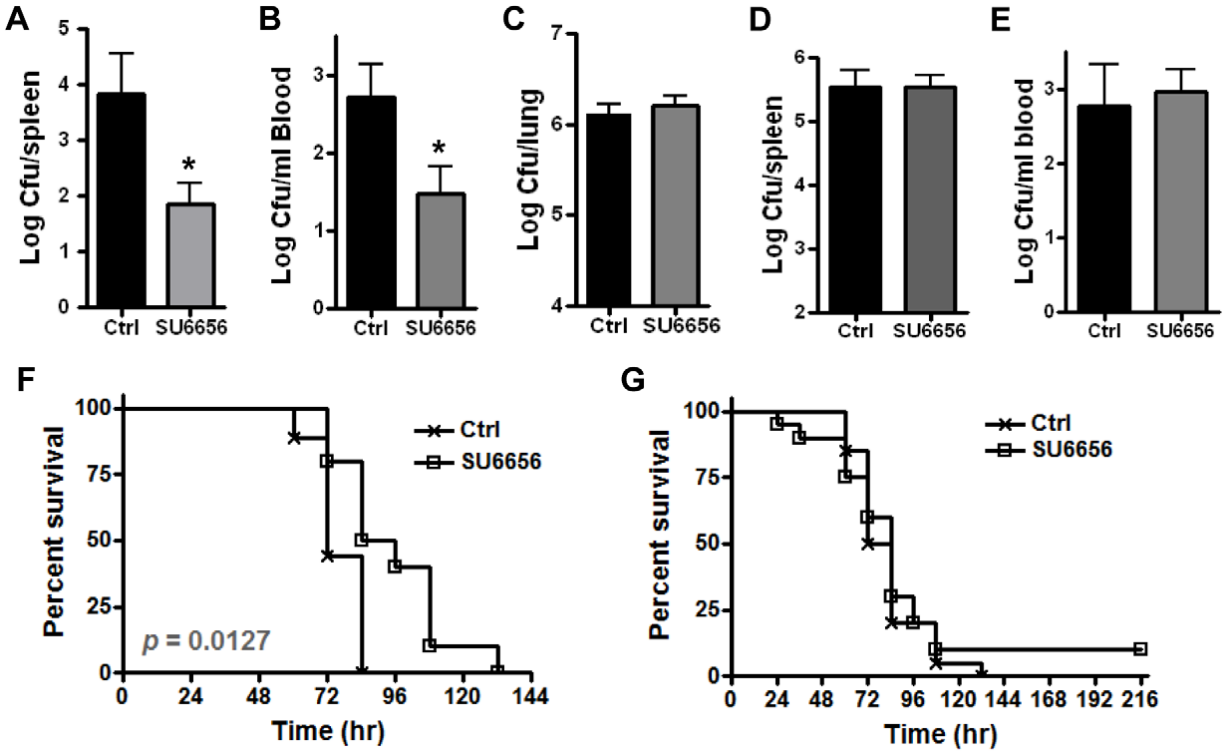
Src activity is important for bacterial dissemination *in vivo*. A/J mice were treated with SU6656 (7.5 µg/g body weight) or equivalent volumes of solvent 24 hr prior to spore inoculation as described in Materials and Methods. **A – C**, bacterial burden in the spleen (**A**), blood (**B**) and lungs (**C**) of mice inoculated by intranasal instillation. Each mouse was inoculated with 1 - 3×10^6^ spores. Organs were harvested at 72 hr post inoculation, homogenized and plated. The results are combined from 2 – 3 independent experiments with a total of 7–15 mice per treatment group. **D**, mice were inoculated with 1×10^5^ spores/mouse by i.p. injection. Bacterial burden in the spleen was determined at 72 hr post inoculation. The results are combined from 2 independent experiments with a total of 12 mice per treatment group. **E**, mice were inoculated with 1×10^4^ spores/mouse by injection into the tail vein. Bacterial counts in the blood were determined at 84 hr post inoculation. The results were combined from 2 independent experiments with a total of 13 mice per treatment group. Statistical significance was calculated using *t* test. *, *p*<0.05. **F**, SU6656 treatment improves survival in i.n. inoculated mice. Mice were inoculated with ∼8×10^6^ spores/mouse intranasally (10 mice per group). Statistical significance was calculated using the Logrank test. Similar trend was observed in three other experiments. **G**, mice were inoculated with ∼1×10^5^ spores/mouse by i.p. injection. The results are from two experiments with a total of 20 mice per group.

In pilot experiments, A/J mice were inoculated with ∼1–3×10^6^ spores/mouse by intranasal instillation (i.n.). Bacterial counts in the spleen were determined at 24, 48 and 72 hr post inoculation. Few bacteria were found in the spleens at 24 or 48 hr. However, substantial amounts of bacteria were recovered at 72 hr. We also found that 72 hr is the approximate median survival time for mice infected with the indicated spore dose via the i.n. route. To determine if inhibition of Src activity would affect bacterial dissemination *in vivo*, mice were treated with either SU6656 (7.5 µg/g body weight) [24] or an equivalent volume of solvent by daily i.p. injection as described in Materials and Methods. Mice were inoculated with spores via the i.n. route and bacterial dissemination was examined at 72 hr post inoculation. Significantly fewer bacteria were recovered from the spleen and blood of SU6656-treated mice compared to those from the control mice (*, *p*<0.05) (Fig. 6, A and B). In contrast, there was no significant difference in the lung bacterial counts between the treated and the control groups (Fig. 6, C), suggesting that SU6656 treatment did not affect bacterial survival in the lung. To further examine whether the reduction in bacterial burden could be due to non-specific effects of SU6656, spores were directly injected into the peritoneum (i.p.) or the tail vein (i.v.) of mice. The appropriate spore dose and the median survival time for the i.p. and i.v. routes of infection were determined in pilot experiments. The spleen bacterial burden in i.p. inoculated mice were essentially the same between SU6656-treated and the control mice at 72 hr post inoculation (∼ median survival time) (Fig. 6, D). For i.v. inoculated mice, blood were collected at 84 hr post inoculation (∼ median survival time) and dilution plated. No difference was observed in the blood bacterial counts between the treated and the control groups (Fig. 6, E), suggesting that SU6656 did not affect bacterial survival or growth in the blood. The effect of the inhibitor on mouse survival was also tested. The results showed that although SU6656 treatment did not prevent death, it prolonged mouse survival time (Fig. 6, F, *p*<0.05, compared with the control group). The median survival time was increased by an average of 12 hr with the inhibitor treatment (84 hr for the treatment group vs. 72 hr for the control group, calculated from 4 independent experiments with a total of 37 mice per group). In contrast, in i.p. inoculated mice, there was no difference in the survival curves between the SU6656-treated and the control groups (Fig. 6, G). Together these results strongly suggest that Src activity is important for bacterial dissemination through the respiratory epithelium and virulence *in vivo*.

## Discussion

The airway and lung epithelium not only functions as a mechanical barrier to inhaled pathogens but also actively participates in host immune responses such as pathogen recognition and production of cytokines, chemokines and anti-microbial peptides. It is thus an important component in the host defense system against microbial pathogens. On the other hand, to establish an inhalational anthrax infection, *B. anthracis* must breach the respiratory epithelial barrier. Earlier studies suggested that *B. anthracis* spores potentially could use lung epithelial cells in addition to AMs and LDCs as portals for dissemination through the barrier [1], [4]. The recent finding that lung epithelial cells rather than macrophages or neutrophils were responsible for the induction of innate resistance to pulmonary exposure of spores highlighted the importance of *B. anthracis*-epithelium interactions in the pathogenesis of this organism. The report that mice immunized with inactivated spores were protected by cellular rather than humoral immunity further emphasized the importance of an intracellular stage during the establishment of anthrax infections [5]. Although a fair amount of information is available on spore-macrophage interactions, there are few previous reports on the molecular mechanisms underlying the interactions between spores and lung epithelial cells.

In this study we sought to determine the molecular events involved in spore entry into lung epithelial cells. The results indicate that spore uptake by epithelial cells is dependent on the actin cytoskeleton and a signaling pathway involving Src, PI3K and Cdc42. Furthermore, we provide evidence that Src activity is important for *B. anthracis* translocation through a barrier of lung epithelial cells in culture as well as dissemination from the lung to distal organs in mice.

The involvement of the actin cytoskeleton in spore internalization is supported by three lines of evidence. Uptake of spores by A549, HeLa and primary hSAECs was virtually abolished in the presence of cytochalasin D. Local F-actin enrichment was observed at spore attachment sites, suggesting a “zipper”-like entry mechanism [25]. Furthermore, spore internalization was specifically inhibited by a DN mutant of Cdc42, but not by DN mutants of Rac1 or RhoA, suggesting that Cdc42 is the major Rho-family GTPase regulating the actin polymerization events during spore entry. The requirement for class IA PI3K activities is supported by the following evidence. Spore internalization was inhibited by two structurally and mechanistically distinct PI3K inhibitors, wortmannin and LY294002, and by a DN mutant of the p85 regulatory subunit of class IA PI3K. In addition, Akt-PH-GFP was rapidly recruited to the spore attachment sites, and the recruitment was reduced in the presence of wortmannin or LY294002. The requirement for Src activity is supported by the inhibitory effects of two distinct SFK inhibitors PP2 and SU6656, and specific siRNA knockdown of Src.

**Figure 7:**
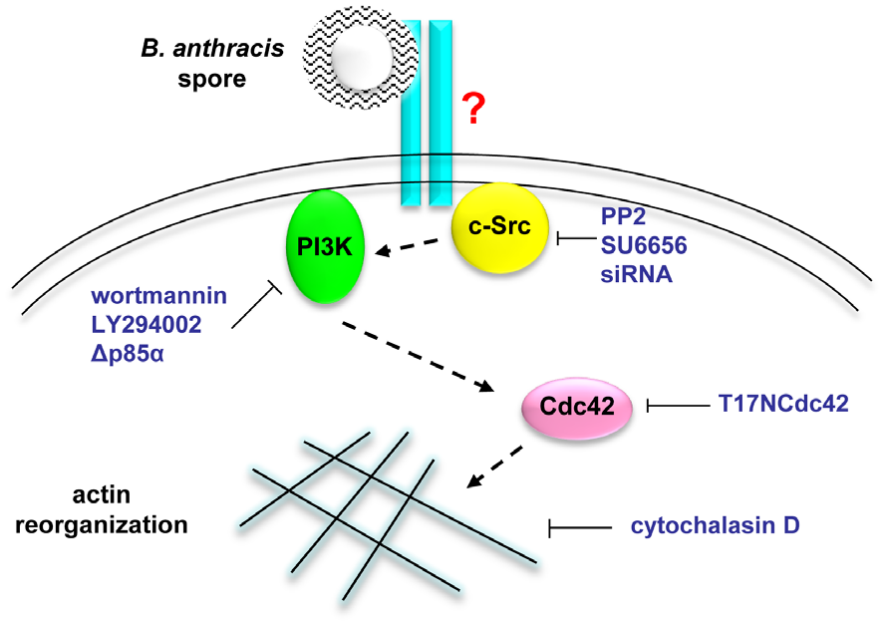
A working model of the mechanism of *B. anthracis* spore internalization by epithelial cells. *B. anthracis* spores interact with an unknown cell surface receptor. The signal is transduced via a pathway including c-Src, class IA PI3K, and the Rho-family GTPase Cdc42. Eventually F-actin polymerization is activated locally around spore attachment sites, leading to spore internalization.

The results also showed that inhibition of either PI3K or Src decreased F-actin enrichment around spore attachment sites. Inhibition of both PI3K and Src did not result in additional decreases of F-actin enrichment as compared to individual inhibitor treatment only, suggesting that PI3K and Src are in the same signaling pathway for spore internalization. In addition, inhibition of Src activity reduced PI3K recruitment and activation, suggesting that Src likely acts upstream of PI3K in the signaling pathway. Based on these results we propose a working model for the signaling pathway responsible for spore entry into epithelial cells (Fig. 7).

The results also provided evidence indicating that the signaling pathway elucidated here is specifically required for spore entry into epithelial cells but not macrophages. This is not surprising considering that the cellular receptors mediating spore entry into these two types of cells are almost certain to be distinct. Oliva *et al*. reported that integrin α_M_β_2_ (Mac-1) was the receptor mediating phagocytosis of wild type spores by macrophages [26]. Since Mac-1 is not expressed in epithelial cells, a different receptor should be required for spore uptake by epithelial cells. Studies to identify the epithelial receptor are currently underway in our laboratory.

The results from the transwell assays indicate that inhibition of Src-mediated spore entry dramatically reduced *B. anthracis* dissemination through epithelial cells. This led us to investigate if inhibition of Src would have a similar effect on dissemination *in vivo*. Although knockout mice are commonly used to examine the importance of a host factor in pathogenesis, *c-src* ^−/−^ mice show a number of abnormalities such as a small body size, a partial absence of bone marrow, abnormal decidualization and in some genetic background post-natal lethality (Jackson Laboratories http://jaxmice.jax.org). Therefore they were not chosen for this study due to the consideration that these abnormalities may complicate the interpretation of results. Instead, we used the SFK inhibitor SU6656. This inhibitor only inhibits four members of the SFK family, Src, Fyn, Yes and Lyn and thus is far more selective than PP1 or PP2, the other two commonly used SFK inhibitors [27]. The results showed that SU6656 treatment at the indicated dosage significantly reduced bacterial dissemination from the lung to the spleen and blood, suggesting that Src activity is important for bacterial dissemination *in vivo*. The observation that SU6656 did not significantly affect bacterial counts in the lungs of i.n. inoculated mice, in the spleen of i.p. inoculated mice, or in the blood of i.v. inoculated mice suggests that under the experimental conditions used in these studies SU6656 treatment does not affect the ability of the host to clear the pathogen, the ability of phagocytes to the pathogen to lymphoid tissues, or pathgoen growth *in vivo*. Thus the reduction of bacterial counts in the spleen and blood of SU6656-treated, i.n. inoculated mice is likely due to a disruption in the dissemination process from the lung to other organs rather than due to non-specific effects of the inhibitor. The SU6656 treatment did not completely abolish dissemination. This is likely due to incomplete inhibition of the kinase activity by the inhibitor and/or dissemination via other routes that do not require this signaling pathway. The effect of SU6656 treatment on mouse survival is consistent with its effect on dissemination. At the dose used, SU6656 significantly improved the survival of i.n. inoculated mice by prolonging the median survival time, whereas it did not affect the survival of i.p. inoculated mice. Death was not prevented since dissemination was not completely blocked. Together these results provide evidence suggesting Src-dependent spore entry into epithelial cells is important for dissemination and virulence *in vivo*. Combined with the previous finding that lung epithelial cells rather than macrophages or neutrophils are responsible for bacterial lysate-induced host resistance to *B. anthracis* [11], the data highlights the need for more studies to understand the molecular details of spore-epithelium interactions and their impact on bacterial pathogenesis and host defense. It should be noted that lung epithelial cells may not be the only cell type affected by SU6656 treatment *in vivo*. Despite all the control animal experiments, the possibility that the effect of SU6656 treatment on dissemination and survival is due to altered activities in other cell types cannot be excluded. Further tests in mouse models lacking the specific epithelial receptor will permit more targeted examination of the complex interplays between the pathogen and the lung epithelium.

That disruption of SFK activities can impair dissemination of microbial pathogens was previously described for *Streptococcus pneumoniae*, although in a somewhat different context. *S. pneumoniae* was shown to migrate across respiratory epithelial cells by hijacking the host transcytosis pathway for transporting polymeric immunoglobulin receptor (pIgR) across polarized epithelial cell [28]. This transcytosis pathway is mediated by the SFK member c-Yes. In *c-yes^−/−^* mice, dissemination of *S. pneumoniae* from the respiratory system to the blood was delayed compared to that in the wild-type mice [28]. Our results from the SFK inhibitor provided another example of interfering bacterial dissemination by inhibiting their entry or passage through epithelial cells. The results also raised the potential of using SFK inhibitors, or inhibitors of other host pathways important for the dissemination of microbial pathogens, as therapeutic agents in conjunction with antibiotics to treat certain infectious diseases.

Another potential importance of spore entry into lung epithelial cells is gaining access to an intracellular niche that may allow persistent colonization of the microbes. *B. anthracis* spores are known to persist in the lung for weeks or months [29], [30], [31], the basis for the 60-day prolonged antibiotic treatment for patients exposed to spores. It was shown previously that spores and vegetative bacilli can survive inside lung epithelial cells for up to 12 hours [1], suggesting that the intracellular environment of lung epithelial cells can potentially be a site for spore persistence. However, it is not known in which intracellular compartments spores and bacilli reside or how long they can remain viable in those compartments. In macrophages, earlier studies showed that after phagocytosis spores were trafficked along the endocytic pathway and eventually were enclosed within LAMP1^+^ lysosomal compartments [32], [33]. How long spores can survive or remain as spores (*i.e.*, not germinate) in the lysosomal compartments of macrophages remains unclear. The fate of germinated spores and vegetative bacilli inside macrophages has been controversial. Some studies showed bacilli replication inside macrophages [32], [33] whereas others indicated efficient killing of vegetative bacilli by macrophages [7], [8]. Therefore it would be interesting to investigate the intracellular events following spore entry into host cells not only in the context of microbial passage through host cells but also persistence within host cells.

Together, the data presented here elucidate a specific signaling pathway required for *B. anthracis* spore internalization by epithelial cells. The pathway includes c-Src, PI3K, Cdc42 and the actin cytoskeleton. The results also demonstrate that this pathway is important for *B. anthracis* translocation through a lung epithelial barrier *in vitro*, dissemination from the lung to distal organs, and virulence *in vivo*. Further studies to elucidate the epithelial receptor(s) with which spores interact and the intracellular events following spore entry will not only be important to the pathogenesis of this organism but will also reveal host receptors and signaling pathways that may be used by other respiratory pathogens to overcome the mucosal barrier.

## Materials and Methods

### Cell culture, bacterial strain and spore preparation

A549 (a human epithelial cell line derived from lung carcinoma, ATCC) and HeLa (a human epithelial cell line derived from cervical cancer cells, ATCC) cells were cultured in F12 and Dulbecco's Modified Eagle Media (DMEM), respectively, supplemented with 10% fetal bovine serum (FBS). Clonetics® primary human small airway epithelial cells (hSAECs) (Cambrex) were maintained at 37°C in a humidified chamber with 5% CO_2_ in SABM media supplemented with reagents from the SAGM SingleQuot kit (Cambrex), following instructions from the supplier. Murine lung epithelial cells (MLE15) were cultured in DMEM with 10% FBS [34]. Murine embryonic fibroblasts (MEFs) from *src-/-yes-/-fyn-/-* triple knockout mice were obtained from ATCC and cultured according to instructions from ATCC. Primary murine peritoneal macrophages from C57BL/6 mice were kindly provided by Dr. Dekai Zhang, Texas A&M Health Science Center – IBT, Houston, TX, and were used within 24 hr of isolation. *B. anthracis* Sterne strain 7702 was provided by Dr. T. M. Koehler, UT Health Science Center, Houston, TX. Spores were prepared from strain 7702 cultured in PA media following a procedure described previously [1].

### Inhibitors and plasmid constructs

Cytochalasin D, wortmannin, LY294002 and SU6656 were purchased from Sigma. PP2 and PP3 were obtained from Calbiochem. All inhibitors were dissolved in DMSO to prepare stock solutions. Plasmids expressing dominant negative p85α (HA-Δp85α) [17], wild-type p85α (HA-p85α) and the vector control (SRα) were provided by Dr. Lei Wei, Indiana University-Purdue University Indianapolis, Indianapolis, IN. Dominant negative constructs for RhoA (HA-T19NRhoA), Rac1 (HA-T17NRac1) and Cdc42 (HA-T17NCdc42) were purchased from UMR cDNA Resource Center (www.cdna.org) [14]. Akt-PH-GFP construct (in pEGFP-N1 vector) was provided by Dr. Tamas Balla, National Institute of Child Health and Human Development, NIH, Bethesda, MD [19].

### Cell transfection

A549 and HeLa cells were transfected with DNA plasmids using Lipofectamine LTX (Invitrogen) following the instructions from the supplier. Briefly, the cells were seeded in 24-well plates at a density of 40,000 cells/well 24 hours prior to transfection. DNA (0.25–0.5 µg) and 0.5 µl Plus Reagent (Invitrogen) were mixed and incubated in 100 µl Opti-MEM I medium (Invitrogen) for 10 min, followed by addition of 1–2 µl of LTX and incubated for 25 min. The mixture was then added to the cells.

Lipofectamine RNAiMAX (Invitrogen) was used to transfect siRNAs into A549 cells following the instructions from the supplier. Briefly, 1 µl RNAiMAX and 100 µl opti-MEM I were mixed with specific siRNAs against c-Src, c-Yes and Fyn (c-Src and Fyn siRNA were from Santa Cruz Biotechnology whereas c-Yes siRNA and the Allstars negative control siRNA were from Qiagen) and incubated for 12 min. The mixture was then added to each well of a 24-well plate to give a final siRNA concentration of 50 nM.

### Western Blot

Cells were harvested 24 hours post DNA plasmid transfection and 48 hours post siRNA transfection, washed and lysed with RIPA buffer (50 mM Tris-HCl pH 7.4, 1% NP-40, 0.25% Na-deoxycholate, 150 mM NaCl, 1 mM EDTA) containing Complete Mini protease inhibitors (Roche). Cell lysates were then mixed with an equal volume of 2× Laemmli sample buffer (BioRad) containing 5% β-mercaptoethanol and boiled at 100°C for 5 min. Samples were subjected to 4%/12% SDS-polyacrylamide gel electrophoresis, and then transferred to a PVDF membrane (Millipore). The membrane was blocked with 5% non-fat milk in a solution of 100 mM Tris-Cl pH 8.0, 0.9% NaCl, and 0.1% Tween20 (TBST), and then incubated with the appropriate primary and secondary antibodies. For detection, the membrane was incubated with a chemiluminescent substrate HyGLO HRP (Denville) for 1 min at room temperature, exposed to X-ray films (Kodak) and developed in a film processor (Konica SRX-101A).

The different primary antibodies were anti-HA (Millipore) (1∶1000 dilution in TBST containing 5% milk), anti-Src (Millipore, 1∶1000), anti-Yes (BD Biosciences, 1∶1000), anti-Fyn (BD Biosciences, 1∶1000) and anti-actin (Millipore) (1∶6000) antibodies. The secondary antibodies were HRP-conjugated goat anti-mouse antibodies (Millipore, 1∶3000) and goat anti-rabbit antibodies (BioRad, 1∶6000).

### Gentamicin protection assay

This was performed as described previously [1] with slight modifications. Briefly, cells grown in 24-well tissue culture plates were incubated with *B. anthracis* 7702 spores at a multiplicity of infection (MOI) of ∼1. The assays were performed in DMEM containing 10% FBS (DMEM/FBS). A549 and HeLa cells were incubated with spores for 1 and 2 hr, respectively. Unbound spores were then removed by washing with PBS. To enumerate associated bacteria (extracelluar adhered and intracellular), cells were then lysed and dilution plated. To enumerate intracellular bacteria, after washing with PBS, cells were further incubated in media containing gentamicin (100 µg/ml) for 1 hour, washed, lysed and dilution plated.

To examine the effect of inhibitors (cytochalasin D, wortmannin, LY294002, PP2, PP3 and SU6656), cells were pre-incubated with the appropriate inhibitor for 1 hr and then incubated with spores in the presence of the inhibitor. For controls, the same amount of the inhibitor solvent was added.

For cells transfected with DNA plasmids, gentamicin protection assays were performed 24 hours post transfection. For cells transfected with siRNA, assays were performed 48 hours post transfection.

### Examine spore adherence and internalization using fluorescence microscopy

This was performed as described previously [1] with slight modifications. A549, HeLa or hSAECs grown on glass coverslips in 24-well plates were incubated with FITC- and biotin-labeled *B. anthracis* spores (MOI of ∼4) in the presence of 2.5 mM D-alanine, a germination inhibitor, for 1 hr for A549 and 2 hr for HeLa and hSAECs. For assays containing inhibitors (cytochalasin D, wortmannin, LY294002, PP2 and PP3), cells were pre-incubated with the respective inhibitor for 1 hr and then incubated with the same amount of labeled spores in the presence of the inhibitors as in the presence of solvent control. Cells were then washed, fixed without permeablization, blocked and incubated with streptavidin – Alexa Fluor 647 (Molecular Probes). After washing, the coverslips were mounted using Fluorsave (Calbiochem) and viewed using a Zeiss Axiovert 135 microscope as previously described [1]. In each experiment, ∼1000 spores in at least 50 fields were counted per test condition.

Spore labeling efficiency was monitored by performing the same procedure as described above but in the absence of cells [1]. Briefly, labeled spores were allowed to attach to poly L-lysine (Sigma) coated coverslips and incubated in DMEM/FBS under cell culture conditions in the presence of 2.5 mM D-alanine. The coverslips were then processed exactly the same way as the coverslips containing cells incubated with spores as described above. Approximately 200 spores were examined for each experiment and the labeling was proved to be efficient.

### Colocalization of F-actin and PI3K with spores

A549 cells grown on coverslips were serum starved overnight and then incubated with Texas Red (Molecular Probes)-labeled spores in DMEM. To examine F-actin colocalization, cells were incubated with labeled spores for 30 min, washed, fixed with 2% paraformaldehyde, and blocked in PBS containing 10% FBS. The cells were then stained with Phalloidin-Alexa Fluor 488 (1∶250 in PBS/FBS, Molecular Probes). To examine recruitment and activation of PI3K, cells were transfected with the Akt-PH-GFP plasmid. 24 hours post-transfection, cells were serum starved and then incubated with labeled spores for 5–15 minutes. Cells were then washed and fixed. The coverslips were mounted and viewed in a Zeiss LSM 510 confocal laser scanning fluorescence microscope with LSM 4.0 software (Zeiss).

For assays involving inhibitors, cells were pre-incubated with the appropriate inhibitor for 1 hr and then incubated with labeled spores in the presence of the inhibitors. In each experiment, ∼100 spores were counted per test condition.

### Cell viability and spore viability

The effect of the inhibitors (cytochalasin D, wortmannin, LY294002, PP2 and PP3) on cell viability was monitored by incubating cells with each inhibitor and the solvent control for the same length of time under the same conditions as in cell infection assays. The cells were then examined by trypan blue exclusion in a haemacytometer. The effect of transfection with various dominant negative constructs on cell viability was also monitored using trypan blue exclusion 24 hours post-transfection. To determine the effect of inhibitors on spore viability, 7702 spores were incubated in the presence of an inhibitor or the solvent control for the same length of time under the same conditions as in cell infection assays and then dilution plated. The number of colony forming units (cfu) from inhibitor-treated spore samples was then compared with that from the control.

### Translocation assays

Translocation assays were performed based on a procedure described previously [1] with modifications. Briefly, A549 cells were grown on collagen-coated polyester Transwell® inserts (Corning) for 13–16 days. DMEM/FBS containing *B. anthracis* 7702 spores (MOI ∼4), 0.1 mM FITC-dextran (Sigma) and 2.5 mM D-alanine were added to the upper chambers and incubated for 16 hours. An aliquot of the bottom chamber media was plated for bacterial counts and another was used to measure fluorescence emission at 520 nm in a LS 50B Luminescence Spectrometer (Perkin Elmer) to monitor the presence of FITC-dextran. The experiment was performed in triplicates and repeated at least twice.

To examine the effect of SFK inhibitors on translocation, cells were pre-treated with PP2 (10 µM), PP3 (10 µM), SU6656 (50 µM), or solvent control for 1 hour. Translocation assays were then carried out in the presence of the inhibitors. The effect of the inhibitors on bacterial growth was examined by incubating 7702 in DMEM/FBS containing the various inhibitors or the solvent at 37°C in a humidified chamber with 5% CO_2_. Samples were collected every hour and dilution plated to determine bacterial counts.

### Mouse infections

All animal experiments were carried out according to procedures approved by the Institutional Animal Care and Use Committee at the Texas A&M Health Science Center, Institute of Biosciences and Technology (IBT) (protocol #08032). A/J mice were originally purchased from the Jackson Laboratory and maintained in the IBT animal facility as approved in the protocol. Mice were 5–8 weeks old when experiments were initiated. For intranasal inoculation (i.n.) of spores, 20 µl of a spore suspension were deposited onto the nares of anesthetized mice to be inhaled. For intravenous and intraperitoneal inoculation, 200 and 100 µl of a spore suspension were injected into the tail vein and the peritoneal cavity of mice, respectively. For inhibitor treatment, approximately 100 µl of SU6656 (the final volume is adjusted to reach a dosage of 7.5 µg/g body weight) or solvent were administered by i.p. injection every 24 hours starting from 24 hours prior to spore inoculation and ending at 48 hours post inoculation. Median survival time (the median time point at which 50% of mice survive) was calculated using the survival analysis tool in the GraphPad Prism 4 program. To examine dissemination, mice were sacrificed, lungs and spleens collected, homogenized and plated to determine bacterial counts. Blood samples were also collected and dilution plated to determine bacterial counts in the blood. Mice were monitored twice daily for the survival studies.

### Statistical analysis

The Logrank test was used to evaluate the statistical significance in mouse survival studies. Student's *t*-test was used to calculate statistical significance in all the other data. The GraphPad Prism 4 software was used for these analyses.

## Supporting Information

Figure S1 PI3K activity is required for *B. anthracis* spore internalization by HeLa cells. The experiments were performed as described in Figure 2 legend. A, spore internalization by HeLa cells was inhibited by WM (50 nM) and LY (50 µM). B, spore adherence to HeLa cells was not affected by WM or LY significantly. C, western blot analysis of the expression of mutant p85α and p85α in transfected HeLa cells. D, spore internalization by HeLa cells was inhibited by the expression of mutant p85α. E, spore adherence to HeLa cells was not affected by mutant p85α.
(0.07 MB PDF)

Figure S2 Src activity is specifically required for spore entry into epithelial cells. A, PP2 (10 µM) was added to cells prior to and during the 1 hour incubation with spores. Spore uptake was assessed following the procedures described in the legends for Figures 1 and 2. Relative uptake was calculated as the percentage of uptake in the presence of PP2 normalized to the no inhibitor control for each type of cells. The results are compiled from at least 3 independent experiments. A549, human alveolar epithelial cell line; HeLa, human cervical epithelial cell line; hSAEC, primary human small airway epithelial cells (Cambrex); MLE, murine lung epithelial cell line MLE15; RAW, murine macrophage cell line RAW264.7; PPM, primary peritoneal macrophages from C57BL/6 mice. B, phagocytosis of spores by RAW264.7 was inhibited by LY294002 (LY). RAW264.7 were pre-treated with LY (50 µM) for 1 hr. Spore phagocytosis was performed using gentamicin protection assays described in Materials and Methods. The phagocytosis assays were performed in the presence of the inhibitor.
(0.04 MB PDF)
