# Supplementary figures and images for: Bacillus anthracis Spore Entry into Epithelial Cells Is an Actin-Dependent Process Requiring c-Src and PI3K
Source: PLoS One. 2010 Jul 20;5(7):e11665. doi: 10.1371/journal.pone.0011665 (PMC2907395; doi:10.1371/journal.pone.0011665)

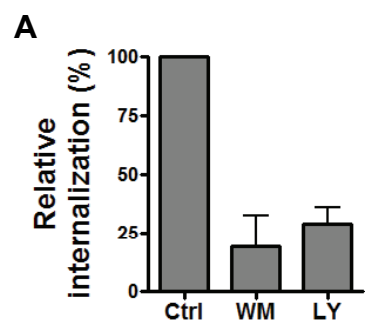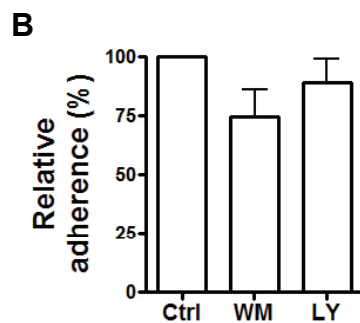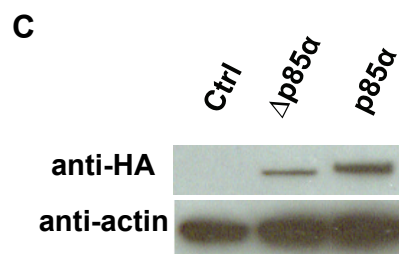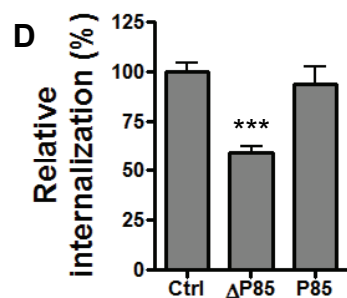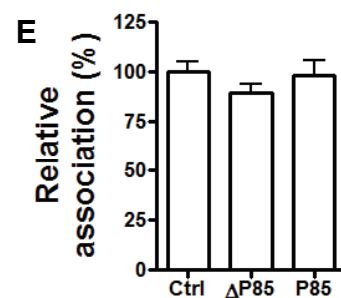

Supplement: Figure S1 — PI3K activity is required for B. anthracis spore internalization by HeLa cells. The experiments were performed as described in Figure 2 legend. A, spore internalization by HeLa cells was inhibited by WM (50 nM) and LY (50 µM). B, spore adherence to HeLa cells was not affected by WM or LY significantly. C, western blot analysis of the expression of mutant p85α and p85α in transfected HeLa cells. D, spore internalization by HeLa cells was inhibited by the expression of mutant p85α. E, spore adherence to HeLa cells was not affected by mutant p85α. (0.07 MB PDF) [file pone.0011665.s001.pdf]

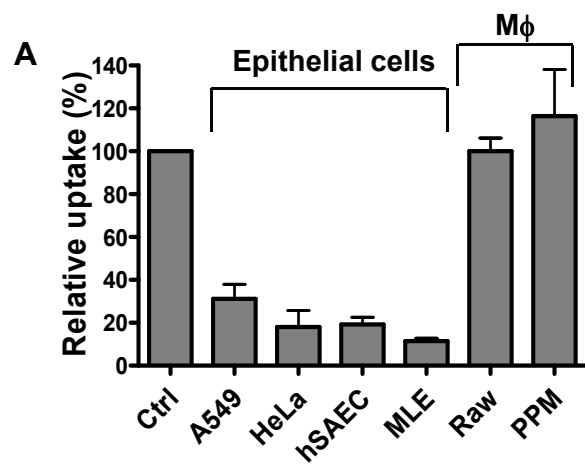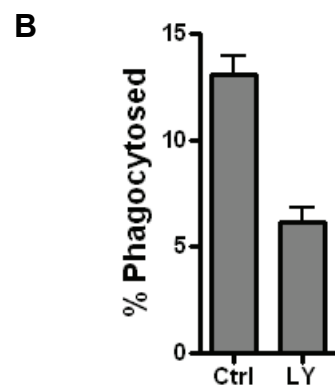

Supplement: Figure S2 — Src activity is specifically required for spore entry into epithelial cells. A, PP2 (10 µM) was added to cells prior to and during the 1 hour incubation with spores. Spore uptake was assessed following the procedures described in the legends for Figures 1 and 2. Relative uptake was calculated as the percentage of uptake in the presence of PP2 normalized to the no inhibitor control for each type of cells. The results are compiled from at least 3 independent experiments. A549, human alveolar epithelial cell line; HeLa, human cervical epithelial cell line; hSAEC, primary human small airway epithelial cells (Cambrex); MLE, murine lung epithelial cell line MLE15; RAW, murine macrophage cell line RAW264.7; PPM, primary peritoneal macrophages from C57BL/6 mice. B, phagocytosis of spores by RAW264.7 was inhibited by LY294002 (LY). RAW264.7 were pre-treated with LY (50 µM) for 1 hr. Spore phagocytosis was performed using gentamicin protection assays described in Materials and Methods. The phagocytosis assays were performed in the presence of the inhibitor. (0.04 MB PDF) [file pone.0011665.s002.pdf]
